# Pancreatic Pseudocyst–portal vein fistula with portal thrombosis and biliary obstruction: a rare complication of necrotising pancreatitis

**DOI:** 10.1093/omcr/omaf211

**Published:** 2026-04-28

**Authors:** Sumanth Srinivasa Shetty, Dhanushan Gnanendran, Lavinia Onos, Rajashekar Gali

**Affiliations:** Department of General Surgery, York and Scarborough Teaching Hospital NHS Foundation Trust, York General Hospital, Wigginton Road, York YO31 8HE, United Kingdom; Department of General Surgery, York and Scarborough Teaching Hospital NHS Foundation Trust, York General Hospital, Wigginton Road, York YO31 8HE, United Kingdom; Department of General Surgery, York and Scarborough Teaching Hospital NHS Foundation Trust, York General Hospital, Wigginton Road, York YO31 8HE, United Kingdom; Department of Interventional Radiology, York and Scarborough Teaching Hospital NHS Foundation Trust, York General Hospital, Wigginton Road, York YO31 8HE, United Kingdom

**Keywords:** pancreatic Pseudocyst, necrotizing pancreatitis, portal vein fistula, biliary obstruction

## Abstract

Background: Pancreatic pseudocyst fistulation into the portal venous system is rare. This may cause portal-vein thrombosis, portal hypertension, and biliary obstruction, complicating diagnosis and treatment. Case: A 45-year-old man with alcohol-induced necrotizing pancreatitis developed jaundice, pain, and abnormal liver tests. Imaging revealed a 6-cm pancreatic head pseudocyst connected to the portal confluence and thrombosed portal and splenic veins. CT showed cavernous transformation and bile duct compression. Pancreatic pseudocyst–portal vein fistula (PPVF) with portal hypertension–related biliary obstruction was diagnosed. The bile duct stricture was stented endoscopically; anticoagulation was withheld due to variceal risk. Symptoms and labs normalized within a week. At 3 months, the pseudocyst regressed, and the bile duct remained patent. Conclusion: PPVF should be considered when portal-vein thrombosis and pancreatic pseudocyst coexist. Characteristic imaging can often obviate invasive confirmation. Early multidisciplinary management, prioritizing endoscopic or percutaneous drainage and selective anticoagulation, allows for safe, effective treatment while avoiding high-risk surgery.

## Introduction

Pancreatic pseudocysts complicate up to 20% of severe pancreatitis, often from alcohol misuse or gallstones [1]. Most resolve or respond to drainage, but rare vascular complications occur. Fistulation into the portal vein, pancreatic pseudocyst–portal vein fistula (PPVF), is exceptionally rare, with fewer than 25 cases reported [2–4].

Two non-exclusive mechanisms are proposed. The first suggests a high-pressure pseudocyst ruptures into a previously normal portal vein; the second postulates peri-pancreatic inflammation induces portal or splenic vein thrombosis, and enzymatic erosion completes fistula formation [2, 5]. PPVF can cause portal-vein thrombosis, cavernous transformation, portal hypertension, and biliary compression, leading to jaundice or variceal bleeding. Early recognition is crucial: MRCP showing identical high T2 signals in pseudocyst and portal lumen is virtually pathognomonic and obviates invasive confirmation [6].

We report a case of alcohol-related necrotizing pancreatitis complicated by a pseudocyst eroding into the portal vein, causing thrombosis, portal hypertension, and biliary obstruction, managed with minimally invasive therapy.

## Case report

A Caucasian male in his 40s with a history of alcohol misuse presented in 2021 with acute necrotizing pancreatitis, managed conservatively. He recovered but was readmitted in 2022 with recurrent pancreatitis and pain. Imaging showed chronic pancreatitis, fatty liver, and evolving splenic vein thrombosis. Anticoagulation was withheld due to hemorrhagic risk. He was lost to follow-up and continued to drink alcohol intermittently.

In early 2024, he re-presented with right upper quadrant pain, early satiety, and jaundice. Labs showed cholestatic liver enzyme elevation and mildly increased pancreatic enzymes; however, he remained stable without GI bleeding. Vascular or biliary complications were suspected.

MRI with MRCP was performed due to abnormal liver function tests. Imaging showed a 6-cm pancreatic pseudocyst at the head/neck, abutting the portomesenteric confluence. The pseudocyst communicated with the portal vein, evidenced by T2-hyperintense fluid extending into the vein, with signal intensity identical to pseudocyst contents. The main portal vein and proximal splenic vein were non-opacified, consistent with extensive thrombosis. Multiple serpiginous collateral veins in the porta hepatis indicated cavernous transformation. MRCP showed moderate intrahepatic biliary ductal dilatation upstream of distal CBD compression, suggesting extrinsic biliary obstruction from pseudocyst or venous collaterals.

**Figure 1:**
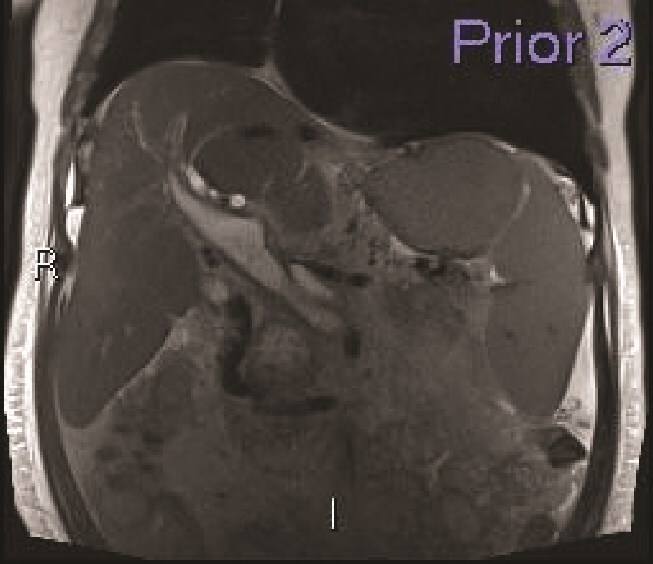
MRI—Coronal reconstruction. The main portal vein and its intrahepatic branches show loss of normal T2 signal void and instead shows distension with T2 hyperintensity within, matching the signal of the pancreatic neck collection (not shown). There is mild bi-lobar intrahepatic biliary radicle dilatation.

**Figure 2:**
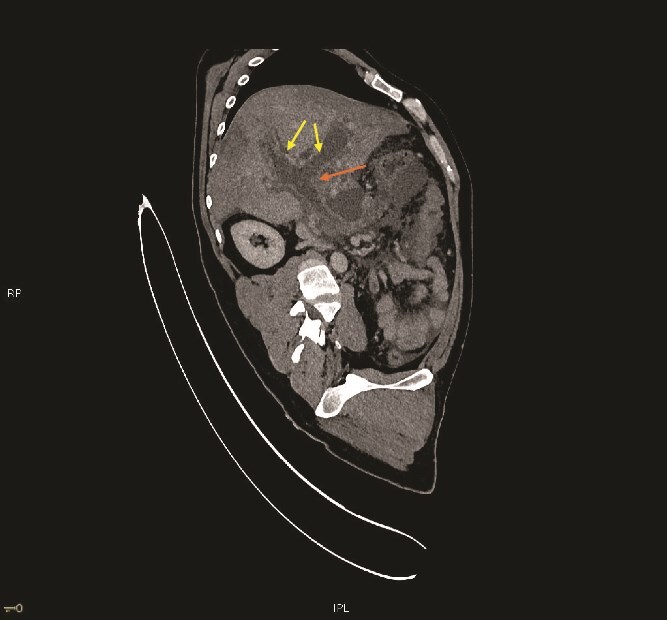
CECT abdomen—Sagittal reconstruction—Pancreatic neck/proximal body shows a fluid collection which is extending into the Porto-splenic confluence (not shown) and causing gross distension of the extrahepatic/main portal vein and the intrahepatic portal vein branches (marked with arrow) with fluid signal matching that of the collection. The main portal vein and its intrahepatic branches show no contrast enhancement. The common bile duct and bi-lobar intrahepatic biliary ducts show moderate dilatation (marked with double arrows). The liver shows heterogeneous enhancement with peripheral wedge-shaped areas of hypo enhancement—Likely as a result of portal hypoperfusion.

**Figure 3:**
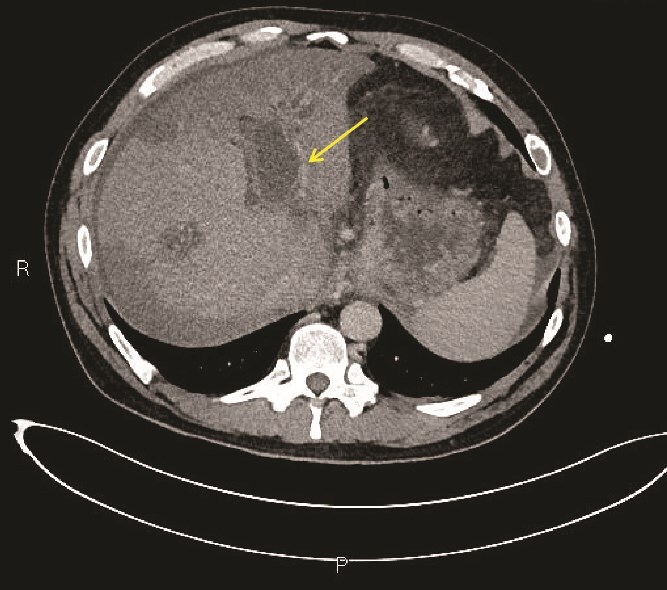
Axial section of CECT abdomen. The intrahepatic portal vein branches (arrow) show gross dilatation/distension with non-enhancing fluid within. The adjacent intrahepatic biliary radicals are dilated due to compression of the common bile duct by pancreatic head collection (not shown). Background liver shows heterogeneous enhancement without features of cirrhosis.

Contrast-enhanced CT confirmed the diagnosis, showing a complex pseudocyst extending into thrombosed portal and splenic veins. The portal vein was filled with low-attenuation material, consistent with thrombus admixed with pancreatic fluid. The splenic vein was also occluded. The liver showed irregular enhancement and small-volume ascites. Splenomegaly and portosystemic collaterals indicated portal hypertension. Intrahepatic and extrahepatic bile ducts were dilated, likely due to pseudocyst mass effect and peri-biliary collateral compression.

ERCP identified a distal CBD stricture, managed with a covered metal stent. Cytology was negative for malignancy. He improved with supportive care; imaging showed decompressed bile ducts and stable pseudocyst. Surgery was deferred due to portal hypertension; EUS-guided drainage reserved for recurrence.

## Discussion

Pancreatic pseudocyst–portal vein fistula (PPVF) is a rare complication of pancreatitis, with fewer than 25 cases reported [2–4]. Although pseudocysts occur in up to 20% of severe cases, PPVF can cause portal vein thrombosis, portal hypertension, and biliary obstruction [3].

Two mechanisms are proposed. The first, ‘thrombosis-first,’ suggests peri-pancreatic inflammation promotes venous thrombosis and wall fragility; subsequent enzymatic erosion by a juxtaposed high-pressure pseudocyst completes the fistula [2, 5]. The second, ‘rupture-first,’ posits an enlarging pseudocyst breaches a normal portal vein, with proteolytic fluid precipitating in situ thrombosis [2, 6]. Only one fatal bleed has been documented after surgery, supporting a model where cystic pressure exceeds portal pressure, driving unidirectional pancreatic juice flow into the vein and rapid occlusion. Our patient’s earlier splenic-vein thrombosis supports the thrombosis-first sequence [5, 7].

Modern cross-sectional imaging allows confident non-invasive diagnosis. On contrast-enhanced CT, a fluid-density, non-enhancing portal vein contiguous with a pancreatic collection is highly suggestive [6]. MRI/MRCP is more specific: an identical high T2 signal in pseudocyst and portal lumen, often with a discernible tract, is considered pathognomonic [6, 8]. ERCP can demonstrate contrast opacification of the portal vein; however, its sensitivity is low, and it is now primarily reserved for therapeutic maneuvers [3].

No consensus management algorithm exists, so therapy is individualized. Conservative observation can suffice in minimally symptomatic patients but fails in up to 60% due to persistent pain, cholestasis, or sepsis. Endoscopic options predominate [4, 9]. Pancreatic duct stenting obliterates the fistula by lowering intraductal pressure, while EUS-guided cystogastrostomy decompresses once the portal vein is occluded [9]. Secondary biliary obstruction, from cyst compression or cavernomatous collaterals (portal biliopathy), is best managed with covered metal stents. Our patient’s rapid biochemical recovery after a covered metal stent underscores this benefit [10]. Anticoagulation in chronic PVT remains contentious; many centers, including ours, withhold it when cavernous transformation and varices are present due to bleeding risk [7, 10]. Surgery is reserved for refractory symptoms or uncontrolled infection/bleeding; pooled case mortality of 15%–20% underscores its role as a last resort [4, 5].

This case adds instructive points to the sparse literature. The combination of PPVF, complete portosplenic thrombosis with cavernoma, and clinically significant distal common bile duct compression is exceedingly rare [2, 3]. Successful management was achieved with single-session ERCP biliary stenting and supportive care, avoiding pancreatic duct instrumentation and surgery. Once the portal vein is occluded, the risk of procedure-related hemorrhage is low, permitting minimally invasive interventions. Long-term follow-up is essential: delayed variceal bleeding, pseudocyst recurrence, and CBD re-stricture have been reported up to 18 months after presentation. Our patient remains under scheduled imaging and endoscopic surveillance.

Limitations include the absence of direct pancreatography visualization of the fistula; however, consensus radiological criteria render invasive confirmation unnecessary when CT and MRI findings are classical [6, 8]. Our follow-up is limited to three months; longitudinal imaging will clarify if further pancreatic or biliary interventions are required. Future multicenter registries are needed to define evidence-based guidance on anticoagulation, optimal timing of EUS drainage, and surgical selection thresholds in this rare but clinically significant complication of pancreatitis.

Pancreatic pseudocyst–portal vein fistula is rare but should be considered if portal vein thrombosis occurs with a nearby pseudocyst. Diagnosis relies on CT/MR showing a fluid-filled portal vein connected to the cyst. Endoscopic or percutaneous drainage is preferred over surgery, and close follow-up is needed for late complications.
